# Asthma in the elderly: a double-blind, placebo-controlled study of the effect of montelukast

**DOI:** 10.1186/s40733-017-0031-4

**Published:** 2017-04-17

**Authors:** Michele Columbo

**Affiliations:** Division of Allergy and Immunology, Bryn Mawr Hospital, Bryn Mawr, PA USA

**Keywords:** Asthma, Elderly, Montelukast

## Abstract

**Background:**

Little is known about asthma in the elderly as most studies of this condition have not included this patient group. It is unclear whether leukotriene antagonists benefit older asthmatics. We studied the effect of adding montelukast to the asthma treatment of elderly subjects.

**Methods:**

Twenty-five subjects 65 years old and older with asthma were evaluated at week 0, 1, 5, 9, 13, and 17. Each subject received montelukast 10 mg and placebo each for 8 weeks in a cross-over design.

**Results:**

Montelukast for 4 or 8 weeks did not significantly affect ACT, daily symptom scores, number of puffs of albuterol, spirometric values, peripheral blood eosinophils, or serum IgE vs. baseline or placebo. Similar results were obtained when analyzing subgroups of patients with lower ACT, lower FEV1, and higher eosinophils.

**Conclusions:**

In this study of elderly asthmatics, montelukast had no effect on asthma symptoms, number of puffs of albuterol, spirometric values, peripheral blood eosinophils or serum IgE. These results will require confirmation in larger patient cohorts and in patients with uncontrolled asthmatic symptoms.

## Background

About 13% of the U.S. population is older than 65 years of age and this percentage is expected to double by the year 2050 [1]. About 7% of individuals 65 years old and older have asthma [1]. However, little is known about asthma in these patients as few asthma studies have examined this patient group. Older patients with asthma are more likely to be underdiagnosed, undertreated, and hospitalized when compared with younger patients affected by this condition [1]. The mortality of older asthmatics is higher than their non asthmatic counterparts [1]. The U.S. National Institute of Aging stated that asthma's pathophysiology is different in the elderly and there are many challenges in the treatments of these patients [1].

Leukotrienes are inflammatory mediators that play a significant role in the pathogenesis of asthma. In particular, leukotrienes are important in bronchoconstriction, airway edema, and mucus formation [2, 3]. They appear to decrease eosinophilic inflammation [4]. Leukotrienes are produced by mast cells, basophils, eosinophils, and other cells [3].

Montelukast (Singulair®) is a leukotriene receptor antagonist US FDA approved for the treatment of asthma in subjects who are 12 months old and older. Its benefits include improved asthma symptoms and spirometric values, and decrease of asthma exacerbations compared to placebo [5]. In general, leukotriene inhibitors are considered less effective than inhaled corticosteroids [6], but they may be beneficial when added to these drugs [7].

There is little information regarding the effect of leukotriene antagonists in elderly asthmatics. Two studies of zafirlukast suggested that this leukotriene antagonist may be less effective in older patients [8, 9]. Two open label studies in subjects older than 60 years old showed that treatment with montelukast may provide clinical benefit when added to inhaled corticosteroids [10, 11].

The main purpose of this pilot study is to investigate the effect of adding montelukast to elderly asthmatics’ treatment regimen on asthma symptoms assessed by the Asthma Control Test (ACT) (primary objective), daily symptom scores, number of puffs of albuterol, and spirometric values. This is a randomized, double-blind, placebo controlled, cross-over study.

Secondary objectives of this study include investigating whether montelukast in elderly asthmatics affects the number of peripheral blood eosinophils and serum IgE levels.

## Methods

This study included six study visits at week 0, 1, 5, 9, 13, and 17. After a one week run-in period, the study subjects were given montelukast and placebo in a cross-over design. They took montelukast 10 mg and placebo each for 8 weeks. Compliance with the study drugs, based on the returned study bottles and with the daily symptom diaries was very good (>90%).

Twenty-five subjects 65 years old and older with asthma completed this study. Twenty-four were Caucasian and 1 African-American. One additional subject discontinued his participation within the first few weeks for reasons unrelated to the study. Allergic sensitization was verified by allergy skin tests for relevant allergens. Current smokers and subjects with > 10 pack/year history of smoking were excluded from this study. Almost all of the study subjects were lifetime nonsmokers. Subjects with recent (<4 weeks before randomization) asthma exacerbation or treatment with systemic steroids, and history of hypereosinophilic disorders were also excluded. Treatment with montelukast or other leukotriene antagonist was not allowed for at least 4 weeks prior to randomization. Study participants were instructed to continue their regular asthma treatment, if any, without any change during the study period unless required by an asthma exacerbation. The study subjects were recruited among interested and eligible patients with asthma followed in a suburban Allergy and Immunology practice.

Spirometric values were obtained according to the ATS/ARS guidelines by a KoKo Spirometer (nSpire Health Inc., Longmont, CO). The ACT is a tool that allows patients to report asthmatic symptoms on a scale from 1 (severe) to 5 (no symptoms) by answering questions about asthma control in the previous 4 weeks. For the duration of the study, each subject filled a daily diary grading their asthma symptoms on a scale from 0 (no symptoms) to 5 (very severe symptoms). The number of puffs of albuterol was also reported daily. Peripheral blood eosinophils were enumerated and total serum IgE measured at the MLH Laboratories at weeks 9 and 17. The inhaled steroids used by the study subjects were fluticasone (14), budesonide (4), mometasone (3), and beclomethasone (1). Inhaled steroid doses are expressed as fluticasone equivalent. Long acting bronchodilators were salmeterol (12), formoterol (5), and vilanterol (1).

Data are expressed as the mean ± standard deviation and analyzed by the two tailed, paired *t* test with significance accepted at <0.05. The number of 25 subjects was calculated as previously reported [12]. In particular, the goal was set to see a 10% improvement (effect size) of the ACT score following the treatment with montelukast vs. placebo. In our earlier study of exhaled nitric oxide measurements in elderly asthmatics [13], the mean ACT score was 22.2 and the standard deviation was 2.8% (*n* = 30). The calculated standardized effect size is 10% of 22.2 (2.2)/2.8 = 0.8. Therefore, β = 0.2 (1/0.8). For an α of 0.05 (two tailed *t* test) the number necessary to see a 10% change is 25.

This study was registered on clinicaltrials.gov (NCT02635334).

## Results

Table 1 summarizes the baseline characteristics of the study subjects. Most subjects were atopic (80%), on inhaled steroids (88%) and long acting bronchodilators (72%), and had well controlled asthma.

**Table 1:** Subjects’ characteristics at baseline

Age (years, range)	72.9 ± 4.9, 66–82
Sex (F/M)	16/9
BMI	26.7 ± 5.6
Atopy	20/25
Duration of asthma (years)	35.7 ± 24.8
Rhinitis	18/25
Gastroesophageal reflux disease	9/25
Inhaled steroids (dose, range)	22/25 (388 ± 324, 0–1,000 mcg/day)
Long –acting bronchodilators	18/25
Anti-cholinergic agents (tiotropium)	5/25
Theophylline	1/25
ACT score	22.9 ± 2.1
Daily asthma symptom score	0.3 ± 0.6
Number of puffs of albuterol per week	0.8 ± 1.8
FEV1%	81.2 ± 15.8
FEV1/FVC	0.74 ± 0.1
FEF25-75%	78.2 ± 31.5
Peripheral blood eosinophils (K/μL)	0.38 ± 0.25
Total serum IgE (IU/ml)	182 ± 249

*N* = 25

Montelukast for 4 or 8 weeks had no effect on ACT (Table 2). At 8 weeks, daily symptom scores and number of puffs of albuterol per week seem decreased by montelukast when compared to placebo (Table 2). However, neither of such decreases was statistically significant. Montelukast exerted no effect on ACT, daily symptom scores, or puffs of albuterol/week when compared to baseline values (*p* > 0.54 for all comparisons).

**Table 2:** Effect of montelukast on ACT, daily symptom scores, and number of puffs of albuterol/week

	Baseline	4 weeks	8 weeks
ACT (montelukast)	22.9 ± 2.1	22.8 ± 2.8	23 ± 3.2
ACT (placebo)		23.4 ± 2.1	22.3 ± 4.4
Daily symptom score (montelukast)	0.3 ± 0.6	0.3 ± 0.5	0.3 ± 0.6
Daily symptom score (placebo)		0.3 ± 0.5	0.5 ± 0.7
Puffs of albuterol/week (montelukast)	0.8 ± 1.8	1.2 ± 3	0.7 ± 1.7
Puffs of albuterol/week (placebo)		0.7 ± 1.5	1.5 ± 3.7

*N* = 25

*p* > 0.3 vs. placebo for ACT; *p* = 0.06 for daily symptom score vs. placebo at 8 weeks; *p* = 0.44 for puffs of albuterol vs. placebo at 4 weeks; *p* = 0.11 for puffs of albuterol vs. placebo at 8 weeks

Montelukast for 4 or 8 weeks did not affect spirometric values when compared to placebo (Table 3). However, The FEV1 values obtained at 4 and 8 weeks for both montelukast and placebo were reduced when compared to baseline (*p* < 0.026 and *p* < 0.052 for montelukast and <0.001 and *p* < 0.002 for placebo, respectively).

**Table 3:** Effect of montelukast on spirometric values

	Baseline	4 weeks	8 weeks
FEV1% (montelukast)	81.2 ± 15.8	77.5 ± 16.1	77.3 ± 17.7
FEV1% (placebo)		76.6 ± 16.4	75.2 ± 17
FEV1/FVC (montelukast)	0.74 ± 0.1	0.75 ± 0.1	0.74 ± 0.1
FEV1/FVC (placebo)		0.74 ± 0.1	0.74 ± 0.1
FEF25-75% (montelukast)	78.2 ± 31.5	75.5 ± 32.2	75.4 ± 35
FEF25-75% (placebo)		72.7 ± 29	73.1 ± 30.8

*N* = 25

*p* > 0.35 vs. placebo for all comparisons

Montelukast for 8 weeks had no effect on peripheral blood eosinophils or serum IgE compared to placebo (Table 4) or baseline values (*p* > 0.34 for all comparisons).

**Table 4:** Effect of montelukast on peripheral blood eosinophils (K/μL) and total serum IgE (IU/ml)

	Baseline	8 weeks
Eosinophils (montelukast)	0.38 ± 0.25	0.37 ± 0.32
Eosinophils (placebo)		0.45 ± 0.42
IgE (montelukast)	182 ± 249	184 ± 272
IgE (placebo)		218 ± 358

*N* = 25

*p* > 0.34 vs. placebo for all comparisons

*Post-hoc* analysis showed that in non atopic subjects, subjects with lower baseline ACT scores (≤21), higher serum IgE (>200 IU/ml), or lower FEV1% (<60%), montelukast did not have a significant effect vs. placebo (Table 5).

**Table 5:** Effect of montelukast in different subjects’ subgroups

	Baseline	4 weeks	8 weeks
ACT nonatopic (montelukast)	22.2 ± 1.8	20.8 ± 4.6	23.6 ± 2.1
ACT nonatopic (placebo)		23.8 ± 0.8	20.4 ± 6
ACT ≤ 21 (montelukast)	20.2 ± 1.8	22 ± 2	21.2 ± 6.4
ACT ≤ 21 (placebo)		21.8 ± 3.3	19.8 ± 7.1
ACT IgE > 200 IU/ml (montelukast)	22.6 ± 2.7	23.5 ± 1.7	22 ± 5
ACT IgE > 200 IU/ml (placebo)		23 ± 3	22 ± 5.4
FEV1% < 60% (montelukast)	54.5 ± 5.4	50.8 ± 9.5	52.2 ± 6.3
FEV1% < 60% (placebo)		50.3 ± 4.9	51.8 ± 6.4
Eosinophils ≥ 0.6 K/μL (montelukast)	0.83 ± 0.22		0.54 ± 0.11
Eosinophils ≥ 0.6 K/μL (placebo)			1.22 ± 0.5

*N* = 8 for ACT IgE > 200 IU/ml; *n* = 5 for ACT nonatopic and ACT ≤ 21; *n* = 4 for FEV1% <60% and eosinophils ≥ 0.6 K/μL)

*p* = 0.07 vs. placebo for eosinophils >0.6 K/μL; *p* > 0.28 vs. placebo for all other comparisons

In subjects with higher baseline peripheral blood eosinophil counts (≥0.6 K/μL), montelukast for 8 weeks appeared to reduce such counts vs. placebo (Table 5), but its effect did not reach statistical significance. In these subjects, asthma symptoms and spirometric values were unaffected by montelukast (data not shown).

## Discussion

In this pilot study, we evaluated the effect of adding montelukast to the treatment of elderly subjects with asthma. This group of patients has been mostly ignored in previous studies of this condition.

Two open label studies in asthmatic subjects 60 years old and older showed clinical benefits from adding montelukast to inhaled corticosteroids with or without long acting bronchodilators [10, 11]. Such benefits included a reduction of asthma exacerbations [10, 11], reduction of the percentage of days with asthma and short beta-receptor agonist [10]. In the present study, adding montelukast for 4 and 8 weeks to our subjects’ treatment had no effect on ACT or spirometric values. Montelukast for 8 weeks appeared to reduce daily symptoms scores and number of puffs of albuterol vs. placebo, but such results did not reach statistical significance, possibly due to the low baseline values. Indeed, a significant limitation of our results is that most study subjects had well controlled asthma. The lack of effect of montelukast on clinical parameters could have been the result of this limitation.

A previous study showed that montelukast decreases peripheral blood eosinophils [6]. Another study failed to detect an effect of montelukast on sputum eosinophils when added to inhaled corticosteroids [11]. In the present study of elderly asthmatics, montelukast did not affect peripheral blood eosinophils or serum IgE levels. Montelukast appeared to decrease eosinophils in subjects with higher baseline blood counts of these cells, but these results did not reach statistical significance possibly due to the small number of observations. Although no clinical benefit of montelukast in these subjects was observed, it can be hypothesized that this subgroup of patients may be more responsive to this drug.

Additional limitations of this study include the relatively small number of subjects who were almost exclusively Caucasian. It is possible that a longer duration of treatment might have yielded different results. It is also possible that montelukast may have a positive effect on other important clinical parameters such as asthma exacerbations and asthma related emergency room visits that could not be assessed in our study.

## Conclusions

In summary, in this study of stable elderly asthmatics, adding montelukast had no effect on asthma symptoms, use of albuterol, spirometric values, or peripheral blood eosinophils. These results will require confirmation in larger studies enrolling subjects with uncontrolled asthma.

## Abbreviations

ACT: Asthma control test
BMI: Body mass index
